# Capturing physiological hemodynamic flow and mechanosensitive cell signaling in vessel-on-a-chip platforms

**DOI:** 10.3389/fphys.2024.1425618

**Published:** 2024-07-29

**Authors:** A. Martier, Z. Chen, H. Schaps, M. J. Mondrinos, J. S. Fang

**Affiliations:** ^1^ Department of Biomedical Engineering, School of Science and Engineering, Tulane University, New Orleans, LA, United States; ^2^ Department of Cell and Molecular Biology, School of Science and Engineering, Tulane University, New Orleans, LA, United States; ^3^ Department of Physiology, School of Medicine, Tulane University, New Orleans, LA, United States

**Keywords:** organ chips, microphysiological systems, vessel-on-a-chip, fluid shear stress, wall shear stress, mechanotransduction, hemodynamic flow

## Abstract

Recent advances in organ chip (or, “organ-on-a-chip”) technologies and microphysiological systems (MPS) have enabled *in vitro* investigation of endothelial cell function in biomimetic three-dimensional environments under controlled fluid flow conditions. Many current organ chip models include a vascular compartment; however, the design and implementation of these vessel-on-a-chip components varies, with consequently varied impact on their ability to capture and reproduce hemodynamic flow and associated mechanosensitive signaling that regulates key characteristics of healthy, intact vasculature. In this review, we introduce organ chip and vessel-on-a-chip technology in the context of existing *in vitro* and *in vivo* vascular models. We then briefly discuss the importance of mechanosensitive signaling for vascular development and function, with focus on the major mechanosensitive signaling pathways involved. Next, we summarize recent advances in MPS and organ chips with an integrated vascular component, with an emphasis on comparing both the biomimicry and adaptability of the diverse approaches used for supporting and integrating intravascular flow. We review current data showing how intravascular flow and fluid shear stress impacts vessel development and function in MPS platforms and relate this to existing work in cell culture and animal models. Lastly, we highlight new insights obtained from MPS and organ chip models of mechanosensitive signaling in endothelial cells, and how this contributes to a deeper understanding of vessel growth and function *in vivo*. We expect this review will be of broad interest to vascular biologists, physiologists, and cardiovascular physicians as an introduction to organ chip platforms that can serve as viable model systems for investigating mechanosensitive signaling and other aspects of vascular physiology.

## Introduction

The blood vasculature is an extensive organ system comprised of hierarchically organized blood vessels that circulate blood from the heart to all tissues of the body. The mechanical forces that blood flow exerts on vascular cells is a key mechanical signal for vascular cell function and homeostasis. Yet, the contribution of hemodynamic flow signaling to healthy and diseased vascular cell function can be difficult to capture experimentally, in part due to the nature of currently available *in vitro* and *in vivo* models of the blood vasculature. Here, we review the importance of hemodynamic flow in the blood vasculature and discuss how increasingly sophisticated microphysiological models of the vasculature, or so-called vessel-on-a-chip systems, may enable new study of flow-sensitive signaling and mechanotransduction in the blood vasculature.

Research on the biology of the blood vasculature has traditionally taken advantage of two general types of model systems. First, many studies use *in vitro* two-dimensional (2D) or three-dimensional (3D) cultures, with the latter often entailing co-culture of endothelial cells with vascular support cells, including fibroblasts, vascular smooth muscle cells, and/or pericytes. The simplicity and high throughput capacity of 2D cultures has enabled discovery of many fundamental aspects of endothelial cell biology, including response to shear stress in 2D cultures of endothelial cells in parallel plate flow chambers (James et al., 1995; Sedlak and Clyne, 2023). 3D cultures in hydrogels composed of natural ECM proteins are useful tools for modeling vasculogenesis and the impact of culture parameters on vascular network architecture. Vascular organoids generated with induced pluripotent stem cell derivatives are powerful tools for modeling key aspects of vascular niche formation including pericyte interactions and basement membrane synthesis (Mondrinos et al., 2014; Wimmer et al., 2019). Collectively, these evolving 2D and 3D culture models have advanced our understanding of vascular biology and the pharmacodynamics of drugs targeting the vasculature (Cochrane et al., 2019; Haas et al., 2020).

Regarding hemodynamics and more complex aspects of vascular physiology, most of the aforementioned *in vitro* cell culture systems fail to simultaneously capture the 3D architecture and flow perfusion of blood vessels *in vivo*. On the other hand, studies of intact blood vessels can be performed either *in vivo* in research animals (e.g., mice, rats, hamsters, *etc.*) or in *ex vivo* blood vessel explants. However, *in vivo* models can be expensive, time-consuming, and potentially incompatible with certain experimental approaches. Modeling the effects of different flow conditions in a reductionist manner while simultaneously controlling for other system elements is challenging unless isolated *ex vivo* vessel preparations are used (Adamson et al., 1994). Furthermore, differences between human and research animal physiology may further complicate interpretation of data for clinical translation (Seok et al., 2013).

In the last 20 years, new *in vitro* models—termed organ chip (or, “organ-on-a-chip”) technology and so-called microphysiological systems (MPS)—have emerged as powerful tools to complement existing *in vitro* and *in vivo* tissue models. MPS, which were originally conceived as “human surrogates” to serve as platforms for modeling pharmacokinetics and pharmacodynamics *in vitro* (Viravaidya and Shuler, 2004; Edington et al., 2018), can fill the gap between traditional culture systems and animal models by situating human cells in a more physiologically-relevant context with controlled biochemical and biophysical parameters (Bhatia and Ingber, 2014).

Early MPS designs focused on the use of printed microfluidic features to interconnect cultures of multiple human cell types and enable real-time communication *via* the transport of soluble factors. In 2004, Viravaidya et al. developed a “microscale cell culture analog” system in which a series of culture chambers containing lung and liver cells were connected by patterned microfluidic channels to “mimic the circulatory system.” (Viravaidya et al., 2004) While in this model an acellular circuit of microfluidic channels accomplishes the essential macroscale transport functions of the circulatory system, the endothelium is in itself an organ that influences tissue-specific and systemic physiology (Augustin and Koh, 2017). Thus, development of systems that faithfully recapitulate the vascular niche of intact blood vessels has long been considered a critical requirement for improving the physiological relevance of MPS (Ewald et al., 2021). In 2010, Huh and colleagues integrated a cellularized vascular compartment in their lung-on-a-chip model of the alveolar-capillary tissue-tissue interface. In this model, a lower tissue channel is lined with primary vascular endothelial cells to form an endothelial monolayer cultured under pump-driven flow and separated from an overlying lung epithelial monolayer by a porous elastomeric membrane that enables inter-tissue exchange of growth factors and nutrients (Huh, 2015). This pioneering microphysiological model of the blood-air interface of the lung has since been used to capture a variety of complex tissue interactions, including immune responses to respiratory virus infection (Huh, 2015; Si et al., 2021).

In the years since these early proof-of-concept prototypes, there has been a proliferation of unique organ chip models—all varying in their platform design and target tissue, as well as presence (or absence) of a vascular tissue component. These include (but are not limited to) working organ chip models of the gut and associated microbiome (Langer et al., 2019; Poceviciute and Ismagilov, 2019), the female reproductive system (Xiao et al., 2017), the pancreatic islet (Bender et al., 2024), the blood-brain barrier (Phan et al., 2017; Campisi et al., 2018), and cancer growth and metastasis (Jeon et al., 2015; Sobrino et al., 2016; Chen et al., 2017; Hachey et al., 2021; Hachey et al., 2024) among many others. Ongoing efforts to engineer body-on-a-chip systems will likely require capturing the physiology of a continuous intact vasculature under physiological flow to model systemic processes and pharmacokinetics (Vogt, 2022). In this review, we will focus on the importance of hemodynamic flow as a regulator of vascular physiology and disease, and explore the challenges of integrating these physiological flow forces in next-generation vessel-on-a-chip designs.

## The blood vasculature and hemodynamic flow

**FIGURE 1:**
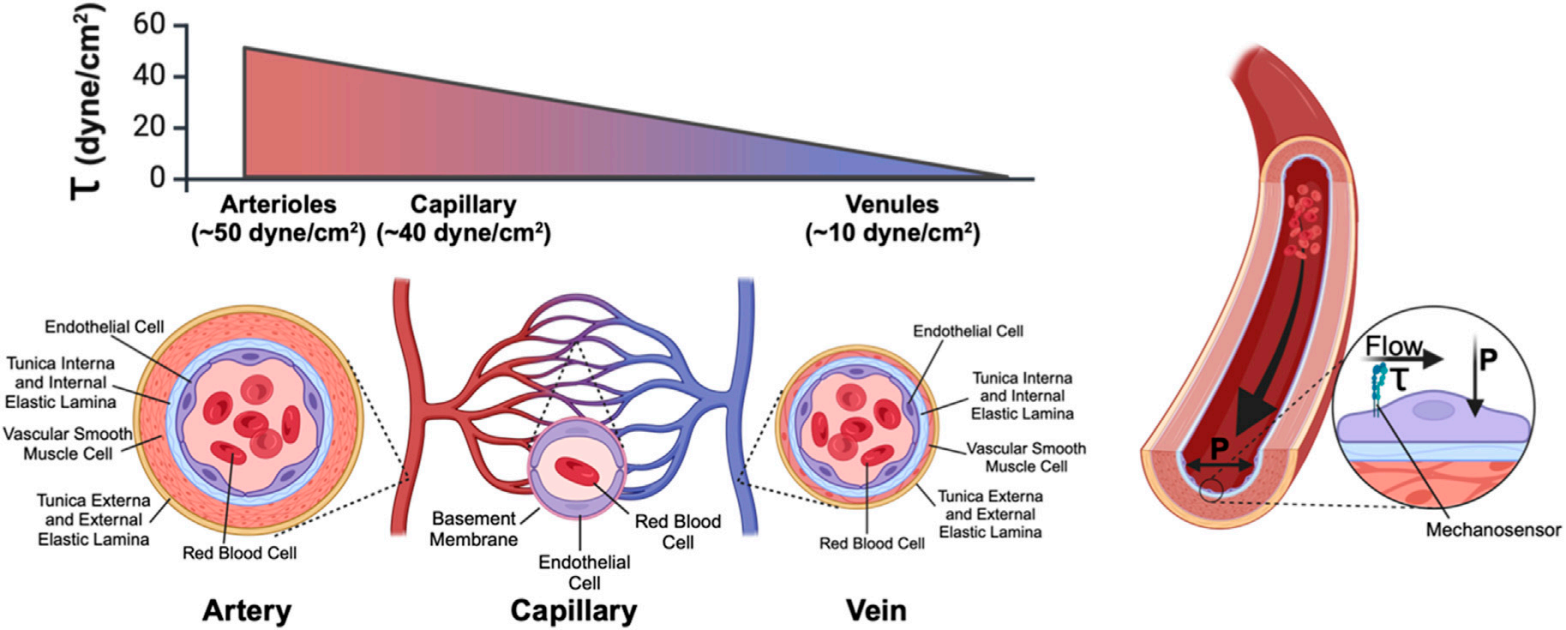
Anatomy of a blood vessel. Blood vessels are lined on their luminal surface by the vascular endothelium, which consists of a single continuous layer of endothelial cells. Surrounding the vascular endothelium is an extracellular matrix layer (tunica interna) which separates the endothelium from the medial layer, which consists of vascular smooth muscle cells (arteries/arterioles, and veins/venules) or pericytes (capillaries). The outermost layer of blood vessels is the tunica externa, another collagen-rich extracellular matrix layer. Within a microvascular network (in which blood flows from arteriole to capillary to venule) fluid shear stress (τ) (the frictional force of fluid flow along the luminal face of the vasculature and that is sensed by endothelial-expressed cell surface mechanoreceptors) is high in the arteriole and low in the venule. Transmural pressure (P), generated by the hydrostatic pressure imparted by fluid in the vessel, acts perpendicular to the endothelium.

Several specialized blood vessel types support efficient systemic circulation, including muscularized arteries and arterioles that constrict and dilate to control local and systemic blood flow; thinly-walled veins and venules that function as elastic capacitance reservoirs for excess blood volume, and intervening small-caliber capillaries (typically 8–10 µm in diameter) that connect the arteriolar and venous sides of the circulatory system and that function as the primary site of oxygen, nutrient, and waste exchange between blood and surrounding tissue. All blood vessels are lined on their luminal surface by endothelial cells—specialized epithelial cell types that form a continuous barrier between circulating blood and surrounding tissue. The apical, lumen-facing surface of vascular endothelium is enriched for proteoglycans and glycoproteins, which forms a protective and immunoregulatory layer known as the glycocalyx that interfaces directly with circulating blood (Figure 1). The basolateral side of endothelial cells, on the other hand, rests upon the tunica interna—a collagen-, laminin-, and fibronectin-rich basement membrane supported by an elastin-rich internal elastic lamina that confers radial elasticity onto the blood vessel. Beyond the tunica interna, perivascular cells—including contractile smooth muscle cells and vessel-stabilizing pericytes—form a medial layer that stabilizes blood vessels and—in the case of smooth muscle—constricts and relaxes to establish local vessel tone and regulate downstream blood flow. On the outer surface of the blood vessel is the tunica externa, a collagen-rich layer of extracellular matrix that in arteries also includes an external elastic lamina that confers additional vascular elasticity to the vessel. Mural cells are physically separated from the vascular endothelium by the tunica interna except *via* pores in the intervening extracellular matrix layer (termed myoendothelial junctions (Heberlein et al., 2009)) that enable endothelial-mural cell-cell contact and intracellular exchange of signaling molecules.

Endothelial cells critically drive both the development and function of the blood vasculature (Garcia and Larina, 2014). During embryonic development, primordial endothelial cells coalesce into a primitive and lumenized vascular plexus (vasculogenesis), which subsequently expands by sprouting angiogenesis to establish new blood vessels in surrounding avascular tissue. Once blood vessels are established in the post-natal animal, vascular endothelium is highly specialized to perform key functions including to regulate blood flow distribution through local control of vessel tone; to mediate vascular-stromal exchange of oxygen, nutrients, and waste; and to regulate inflammation and immune cell responses. Under some physiological (e.g., menstruation) and pathological (e.g., cancer neovascularization, wet age-related macular degeneration, diabetes, *etc.*) conditions, the vascular endothelium may also reactivate pro-angiogenic signaling pathways to grow new blood vasculature.

As the interface between circulating blood and surrounding tissue, vascular endothelium is exposed to hemodynamic flow that exerts (and is thus sensed by primary mechanosensory cell surface receptors (Figure 1) as) mechanical friction forces (i.e., wall shear stress, WSS or τ) onto the lumen-facing (apical) surface of endothelial cells. Local WSS depends upon both vessel diameter and the flow characteristics of circulating blood. WSS can be calculated (Papaioannou and Stefanadis, 2005; Khankin et al., 2021) as:

$$WSS=4ƞQ/\pi r^{3}$$

In this equation, ƞ is blood viscosity, Q is fluid velocity, and r is vessel diameter—all are critical parameters that determine local WSS. Importantly, of these parameters, both fluid velocity and vessel diameter can vary significantly across the systemic circulation. While fluid velocity is directly correlated with shear, this equation underscores how even small changes in vessel diameter—such as what occurs in the hierarchal branching of the vascular tree—can produce large effects in WSS (Table 1). Endothelial cells of the aorta, for example, experience high-velocity pulsatile flow at magnitudes of ∼20 dynes/cm^2^ (∼2Pa) (Callaghan and Grieve, 2018). Pulsatile flow is defined as flow that oscillates between periods of high and low pressure, which corresponds to systole and diastole; flow pulsatility is quickly lost as the vessel wall dampens the pressure oscillations leading to steady downstream laminar flow. By contrast, WSS values for the microvessels are on average 40 dynes/cm^2^, but flow through these vessels is intermittent due to dynamic, moment-to-moment vasomotor changes that alter precapillary vessel diameter to control whether downstream capillary networks are perfused. Shearing forces are further compounded by the movement of circulating red blood cells as they squeeze through—and thus drag along the inner wall of—small caliber capillaries (Figure 1). Thus, capillary flow and WSS can vary by as much as 3–96 dynes/cm^2^ (0.3Pa–9.6Pa). Importantly, normal vascular WSS values are also species-specific: mice, for example, can experience WSS of ∼600 dynes/cm^2^ in the aorta, whereas such WSS forces are rarely found in humans (Suo et al., 2007).

**TABLE 1:** Flow characteristics and average wall shear stress forces in human vasculature.

	Flow type	Wall shear stress (dynes/cm^2^)	Reference
Aorta	Pulsatile	5–22	Callaghan and Grieve (2018)
			Chatterjee (2018)
Artery	Laminar	3–13	Reneman and Hoeks (2008)
			Chatterjee (2018)
Arteriole	Laminar	10–60	Reneman and Hoeks (2008)
			Hoesseinzadegan and Tafti (2017)
			Chatterjee (2018)
Microvessels	Intermittent	28–955 (Average: 40)	Koutsiaris et al. (2007)
Venule	Laminar	10	Ballerman et al. (1998)
			Chatterjee (2018)
Vein	Laminar	1–5	Ballerman et al. (1998)
			Chatterjee (2018)

^a^ All values are approximate ranges based on referenced studies and reviews.

WSS is a key biophysical signal for endothelial cells, and influences many aspects of vascular biology by altering endothelial cell shape, proliferation (Guo et al., 2007; Fang et al., 2017), migration (Hsu et al., 2001), gene expression and signaling activation (Guo et al., 2007; Fang et al., 2017; Chung et al., 2022; Mendez et al., 2022), and junctional permeability (Baeyens et al., 2016). During blood vessel development, WSS is a critical signal for proper vascular morphogenesis, and onset of systemic blood flow from the heart drives reorganization, remodeling, and mural cell recruitment to transform this plexus into a mature vascular network that includes arteries, capillaries, and veins (Lucitti et al., 2007; Garcia and Larina, 2014). For example, exposure of endothelial cells to arterial levels of flow drives expression of the arterial identity gene Cx40, and knockout of this gene leads to disrupted arteriogenesis (Buschmann et al., 2010; Fang et al., 2017). By contrast, vascular malformations in the congenital disease Hereditary Hemorrhagic Telangiectasia (HHT) occur primarily in high flow vessels (Larrivee et al., 2012), and appear to arise from defects in flow-sensitive endothelial cell migration (Jin et al., 2017). In post-natal vasculature, healthy vascular endothelial cell function remains tightly regulated by flow, especially in regard to vascular barrier function where flow induces profound changes in expression of junctional proteins [e.g., cadherins (Miao et al., 2005), gap junctions (Fang et al., 2017), and tight junctions (Yang et al., 2020)]. Flow also controls cell-cell signaling [e.g., Notch (Fang et al., 2017; Mack et al., 2017)], activates vasodilatory signals [e.g., eNOS (Sahni et al., 2023)], suppresses anti-inflammatory KLF2 signaling and increases production of circulating cytokines (Fledderus et al., 2007; Chen et al., 2021), and induces endothelial cell cycle arrest and specification (Fang et al., 2017). Abnormal WSS signaling is associated with endothelial cell dysfunction, and is a major contributor to the pathophysiology of vascular diseases such as atherosclerosis (Zhou et al., 2023). In the lymphatic circulation, WSS is similarly critical for lymphatic vascular development and function (Angeli and Lim, 2023).

Notably, endothelial cells are exposed to transmural blood-pressure mediated hydrostatic pressure (P, Figure 1) in addition to WSS. The innate pulsatile flow of blood throughout the cardiovascular system imparts a significant force against vascular walls. Like WSS, this additional pressure is a known modulator of endothelial cell behavior, both on the individual and tissue scales. Externally applied hydrostatic pressure in culture models has been shown to regulate endothelial cell proliferation, focal adhesion complexes, and integrin expression (Schwartz et al., 1999; Prystopiuk et al., 2018). Tuning the applied hydrostatic pressure to match known physiological ranges protects against barrier damage in endothelial perturbation studies (Muller-Marschhausen et al., 2008). Moreover, hydrostatic pressure has been shown to induce angiogenesis *via* YAP1 signaling in damaged lung tissues (Mammoto et al., 2022) and to promote vascular tube formation *in vitro via* Ras-ERK signaling (Yoshino et al., 2020). Hence, hydrostatic pressure in the vascular system is an important mechanical signal sensed by the endothelium *in vivo* and within the context of *in vitro* systems. Controlling and monitoring hydrostatic pressure should be considered along with applied shear stress in the design and implementation of vessel-on-a-chip systems. Technology for studying the effects of hydrostatic pressure relevant to vessel-on-a-chip systems include microfluidic culture devices with embedded pressure sensors designed to grant independent control of applied pressures and shear stresses (Liu et al., 2013). This review focuses on modeling and interrogating endothelial mechanosensing of shear forces associated with applied fluid flow.

## Diversity in flow-sensitive signaling across endothelial cells

Endothelial cells exhibit distinct responses to the magnitude of WSS forces, indicating exquisite mechanosensing capabilities. Cleavage of the Notch intracellular domain, for example, is maximal in endothelial cells at 18 dynes/cm^2^, but reduced at either higher or lower shear, or under static conditions (Fang et al., 2017). By contrast, Smad1/5 phosphorylation occurs at low (1 dynes/cm^2^) shear stress, but this is suppressed at higher (3 dynes/cm^2^) shear (Mendez et al., 2022). In microfluidic channel models of the vasculature, only application of fluid flow above a minimum threshold induces angiogenic sprouting in an MMP1-dependent manner (Galie et al., 2014). Taken together, the findings that many mechanosensitive signals respond differently to different magnitudes of flow have led to the hypothesis that distinct endothelial cells maintain themselves at a specific fluid shear stress “setpoint” (Baeyens et al., 2015; Baeyens et al., 2016), and that this may be established in an organotypic and vessel-specific manner. Furthermore, the homeostatic regulation of setpoint signaling appears to be a key determinant for endothelial cell biology in health and disease (Baeyens et al., 2015), and may moreover differ across distinct endothelial cell subtypes. Indeed, recent next-generation sequencing analysis of the murine vasculature reveals profound heterogeneity in endothelial cell identity and gene expression (Kalucka et al., 2020), suggesting that fluid shear stress “setpoint” and mechanosensitive responses may also vary widely within an individual blood vessel, between blood vessels of different identity and caliber within a single network, and across distinct tissue-specific blood vasculatures. Lastly, heterogeneity of the endothelium is also heavily influenced by tissue-specific paracrine and ECM signals that function in concert with hemodynamic signals to confer local phenotypes (Aird, 2012; Gunawardana et al., 2021).

Several cell surface proteins have been reported to function as primary mechanosensors that sense and transduce WSS signals into biological cell signaling responses (Figure 1). Of these, the junctional complex comprised of VE-Cadherin/CD31/VEGFR2/3 (Tzima et al., 2005; Coon et al., 2015) has been well characterized and plays a critical role as a mechanosensor of WSS in endothelial cells. The non-selective Piezo1 cation channel (Hyman et al., 2017), Notch1 (Mack et al., 2017), S1PR1 (Cantalupo et al., 2017), and primary cilia structures have also been proposed to be flow mechanosensors (Luu et al., 2018), although how and under what circumstances these mechanosensory proteins and cell structures (individually and collectively) translate WSS signals to control endothelial cell biology in health and disease remains the subject of active research. The ability to precisely control and measure applied shear stress in vessel-on-a-chip systems can allow researchers to investigate shear sensing mechanisms in a physiologically relevant format using human cells. In summary, efforts to mimic the physiology of living blood vasculature in microphysiological systems will require careful consideration of applied flow and the resultant shear stresses that regulate endothelial cell forms and functions.

## History of vessel-on-a-chip systems

**FIGURE 2:**
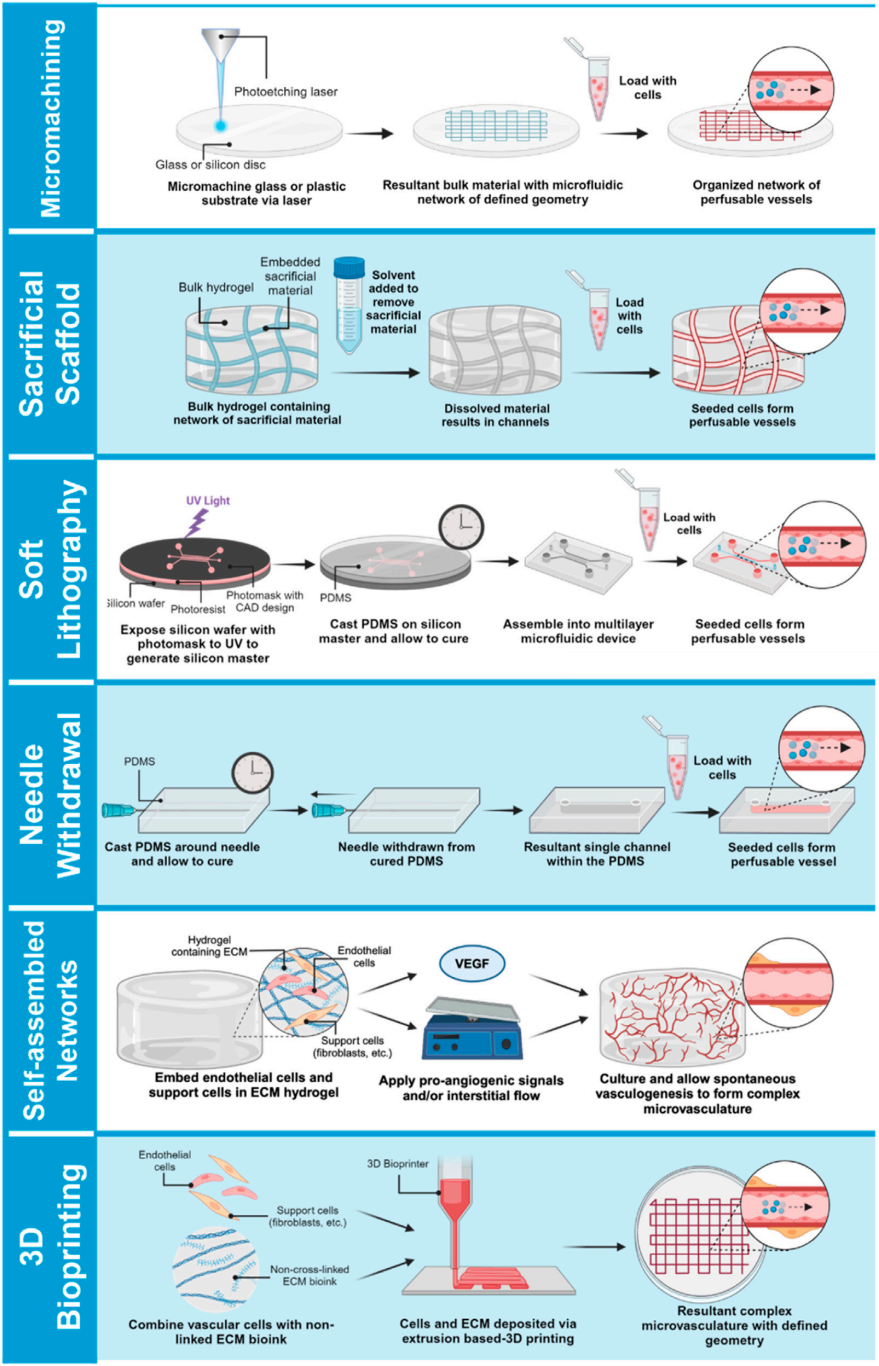
Approaches for bioengineering vessel-on-a-chip models. Micromachined or acellular gel-imprinted microfluidic channels circulate flow between distinct tissue compartments. Alternatively, complex microfluidic channel designs can be formed in a 3D hydrogel by soft lithography printing, or by a needle withdrawal method to generate a single-channel vessel-on-a-chip. Microvessel networks can be formed in organ chips either by embedding endothelial cells with support cells into a 3D hydrogel and inducing network self-assembly by application of interstitial flow, or vessel networks can be bioprinted onto a minimal scaffold, and then exposed to interstitial flow to promote lumenization.

Early efforts to harness microfluidic technologies for vascular engineering largely focused on integrating an artificially engineered circulatory system into bulk-engineered tissue constructs. Methods of patterning a template of channels included micromachining a series of channels in a rigid polymer bulk, or by manipulating the 3D architecture of more compliant extracellular matrix-derived substrates (Figure 2). In the latter approach, a sacrificial material (often made of sugar, alginate, or gelatin) was embedded within a bulk hydrogel in the pattern of the desired vascular structures. Subsequent dissolution of this matrix produced a network of perfusable channels to enable fluid circulation (Golden and Tien, 2007; Bellan et al., 2009; Norotte et al., 2009; Miller et al., 2012; Zervantonakis et al., 2012; Wang et al., 2014). Alternatively, photolithography-based methods could be used to generate micropatterned hydrogels with internal channels approximating vascular structures (Shevkoplyas et al., 2003; Moon et al., 2009; Du et al., 2011; Zheng et al., 2012) Further engineered efforts combined both micromachining and 3D matrix micromolding (Bertassoni et al., 2014). Overall, while such models were able to generate channel networks to perfuse bulk tissues, these printed channel designs were often not endothelialized and lacked many key biological characteristics such as structural heterogeneity of the native vasculature and the presence of stromal support cells.

In recent years, considerable efforts have been dedicated to engineering MPS models of the vasculature, either as a stand-alone vessel-on-a-chip platform or in the presence of organ-specific parenchymal cells (Torisawa et al., 2014; Huh, 2015; Blundell et al., 2016; Jang et al., 2019; Poceviciute and Ismagilov, 2019). Contemporary vessel-on-a-chip platforms vary widely in their design and ability to recapitulate aspects of the intact vascular niche (Table 2). A commonly used single vessel-on-a-chip platform involves fabrication of an extracellular matrix-comprised hydrogel cast within a mold fabricated from polydimethylsiloxane (PDMS) in the presence of a stainless steel needle or similar template (Figure 2). Subsequent withdrawal of the needle produces a templated channel that can then be seeded with endothelial cells to form a complete circumferential monolayer on the inner surface of the channel (Chrobak et al., 2006; Price et al., 2008; Seto et al., 2010; Wong et al., 2010; Sadr et al., 2011; Kusuma et al., 2013; Linville et al., 2016; Polacheck et al., 2017; Polacheck et al., 2019). This approach produces perfusable vessels as small as 160 µm in diameter (Polacheck et al., 2017; Polacheck et al., 2019)—within the range of small peripheral vessels in humans. Linville and colleagues showed that controlled patterns of interstitial pressure, stiffer hydrogel matrices, and high cAMP can enhance the generation of small diameter biomimetic vessels *via* this method (Linville et al., 2016). Hypothesis-driven studies using the needle withdrawal model have yielded insights into endothelial cell signaling mechanisms that control vessel barrier function (Wong et al., 2010; Polacheck et al., 2017; Polacheck et al., 2019). However, this vessel-on-a-chip is limited by difficulties in incorporating a well-organized outer mural cell layer. The single vessel format with constant diameter also lacks the morphology of fractal branching and tapered diameters that contribute to heterogenous flow patterns and gradients of WSS that are present in native vascular beds (Saqr et al., 2020).

**TABLE 2:** Characteristics and Functions of *In Vivo* Vasculature and Examples of their Representation in MPS.

*In Vivo* vascular characteristic	Example(s) of representation in MPS	Reference
Vasculogenesis	Endothelial cells are induced to self-organized into an interconnected vascular network	Campisi et al. (2018)
Sprouting Angiogenesis	Established vessel networks are induced to sprout into an adjacent avascular hydrogel	Chan et al. (2012), Kpeli et al. (2024)
Vessel lumenization	Establishing an empty tube in hydrogel (e.g., by viscous finger patterning or other approaches), which is then endothelialized on the inner luminal surface	Bischel et al. (2012), Polacheck et al. (2019)
Hierarchical and branching network organization	Endothelial cells are induced to self-organized into an interconnected vascular network; microvessels are patterned onto a bioprinted extracellular matrix comprised of a branching architecture	Sobrino et al. (2016), Kolesky et al. (2014)
Vessel barrier maturation	Establishment of an endothelialized tube to study transmural transport; Integration of neural cell types to model the barrier function of the blood-brain barrier	Polacheck et al. (2019), Campisi et al. (2018)
Perfusion and blood flow	Gravity-driven or motorized circulation of media through lumenized vessels	Figure 2, and associated text
Basement membrane formation	Porous elastomeric tissue-tissue interfaces to provide physical separation; artificial ECM analogs	Huh (2015)
Mural cell coverage, i.e., pericytes, smooth muscle)	Vascular mural cells co-seeded with endothelial cells	Sobrino et al. (2016), Campisi et al. (2018)
Conducted vasomotor changes	None reported	
Tissue-specific endothelial cell identity	Co-seeding with tissue-specific EC or stroma derived either from donor tissue or iPSC.	Campisi et al. (2018)
Immune cell transmigration	A chemotactic gradient drives immune cell extravasation across a bioengineered vessel wall	van Os et al. (2023)
Cancer cell metastasis	Cancer cells are circulated in the vascular compartment to measure vascular transmigration	Ozer et al. (2023)

Organ chip models of entire vascular beds have been developed in attempts to create more biomimetic systems for investigating vascular physiology, drug delivery, and pathophysiological processes such as tumor cell metastasis (Haase et al., 2020; Del Piccolo et al., 2021; Chen et al., 2023) (Figure 1). In brief, human donor-derived (i.e., primary or iPSC-derived) endothelial and vascular support cells (e.g., fibroblasts, pericytes, vascular smooth muscle) are co-seeded in extracellular matrix hydrogels loaded in organ chip devices. These organ chips typically entail a “3-lane” design in which a central channel loaded with the 3D hydrogel mixture containing vascular cells is flanked by side channels that can be seeded with a confluent endothelial layer to aid anastomosis with the internal bulk vasculature (Wang et al., 2016). Various approaches have been reported to enhance anastomosis, such as the subsequent application of pro-angiogenic signals (e.g., exogenous VEGF) and/or interstitial flow that drives cytoskeletal rearrangement and increases nitric oxide (NO) synthesis, (Kim et al., 2016). Collectively, numerous approaches can facilitate the self-organization of vascular cells into a patent and perfusable microvascular bed, although defining the mechanism of anastomosis to achieve greater control will require further investigation. Most importantly, the resultant models exhibit organotypic vascular organization (i.e., appropriate lumen size, branching architecture, mural cell wrapping) and grant the ability to precisely control biophysical and biochemical signals for tuned mimicry of *in vivo* blood vessels (Nakatsu and Hughes, 2008; Newman et al., 2011; Chan et al., 2012; Kim et al., 2013). Reports of microvasculature-on-a-chip models outperforming conventional 2D and 3D models in their recapitulation of *in vivo* drug responses are a testament to the translational potential of these platforms (Hachey et al., 2021; Hachey et al., 2024).

Organ chip models of vascular beds that rely on vascular cell self-organization may be difficult to standardize due to variations in cell sourcing, purity, and quality. Recently, some groups have incorporated cutting-edge 3D bioprinting approaches to circumvent this requirement by artificially depositing vascular cells into pre-determined architectures within an extracellular matrix (Kolesky et al., 2014; Gao et al., 2017; Zhang G. et al., 2020; Zhang et al., 2021; Orellano et al., 2022; Zhang et al., 2022), and these models are capable of generating perfused vascular networks supported by minimal scaffolding material (Kolesky et al., 2014; Lee et al., 2014; Jia et al., 2016; Cao et al., 2019; Freeman et al., 2019; Gold et al., 2021). Unlike other models described thus far, bioprinted vessel characteristics are dependent on the mechanical properties of both the bioinks used as well as the surrounding hydrogel scaffold. For example, changing flow rate and print-head speed affects resulting bioprinted vessel lumen size (Attalla et al., 2016). Inclusion of solid frames within the hydrogel scaffold produces tensile forces that also influence vessel morphology (Zhang G. et al., 2020; Zhang et al., 2021; Zhang et al., 2022). While bioprinted platforms have the potential to precisely control 3D architecture, the complexity and technical demands of the approach currently limits it is broad adoption by basic scientists and preclinical researchers. Organ chip models and MPS allow researchers to utilize familiar 2D and 3D cell culture methods using fluidic device platforms with increasing commercial availability as “off the shelf” products to a wide-range of end users (Zhang and Radisic, 2017).

As with all MPS, engineering organ-specific vessel-on-a-chip models require precise control of cell type, microtissue architecture, extracellular matrix composition, and mechanical forces such as substrate stiffness and the shear stress applied by microfluidic flow. Importantly, these parameters are key signals for microvessel-on-a-chip network formation—which underscores the importance of these signals in the function of native vasculature—and are critical for capturing endothelial cell physiology in an *in vitro* setting. In a recent study by Hatch et al. to develop a COVID-on-a-chip vascular model, for example, endothelial cell expression of SARS-CoV2 target ACE2 was reported to be flow-dependent, and a SARS-CoV2 pseudovirus failed to efficiently infect primary endothelial cells except within a vessel-on-a-chip platform under flow (Hatch et al., 2024).

Optimization of chip biomaterials, organotypic cell combinations, extracellular matrix composition is a unique tissue engineering problem within each specific organ chip application. Most organ chips are fabricated out of optically transparent, biocompatible elastomers—most commonly PDMS. Pure PDMS has a relatively low elastic modulus (1.3–3 MPa) and tensile strength (3.5–5.1 MPa) (Ariati et al., 2021), meaning that it is a relatively soft and elastic elastomer when compared to other inorganic materials. Furthermore, the mechanical properties of PDMS can be controlled either by altering the curing conditions of pure PDMS or by including fillers to create PDMS composites that can vary widely in their mechanical properties (Ariati et al., 2021). Nonetheless, pure PDMS is inherently less elastic and more stiff than biological tissues (including intact vessel wall), and most PDMS composites increase—not decrease—these properties (Ebrahimi, 2009). The difference in PDMS stiffness relative to intact basement membrane will affect the resulting shear stress that is generated by fluid flow and sensed by endothelial cells—particularly with regard to organ chips in which endothelial cells are seeded directly onto PDMS-based (or other elastomeric) surfaces and where the PDMS provides direct mechanical support for the engineered endothelium.

Alternatively, blood vessels can be generated within extracellular matrix hydrogels seeded into microfluidic tissue compartments, such that the PDMS of the tissue chamber is not directly in contact with engineered blood vessels and is not directly contributing to mechanical wall stiffness. In these types of vessel-on-a-chip designs, the elastic modulus of vascular mural cells and surrounding hydrogel typically falls within the compliant range of soft tissues—i.e., 1 kPa or less—providing a more physiologically relevant microenvironment that better approximates the mechanical properties of intact blood vasculature. As has been previously shown to be the case in simpler 3D angiogenesis models (Newman et al., 2011), the presence of fibroblasts is essential for network formation. In the absence of perivascular support cells, networks fail to form or regress rapidly (Nakatsu et al., 2003; Newman et al., 2011; Whisler et al., 2014). More research is needed to determine the optimal types and ratios of support cells to produce coveted organ-specific endothelial phenotypes. Extracellular matrix protein composition and density also affects vascular network formation and maturation, likely through combined effects on cell-matrix signaling and matrix stiffness. For example, increased fibrinogen concentration increases the average diameter of resulting vessels (Whisler et al., 2014), and extent of gelatin methacrylate methylation dictates vessel outgrowth potential and vasculogenesis (Chen et al., 2012). Externally applied mechanical load alone also controls vessel architecture (Krishnan et al., 2008; Rosenfeld et al., 2016), suggesting that both cell-matrix signaling and mechanical properties of the extracellular space contribute to vascular morphogenesis in these models.

In summary, MPS research has delivered the foundation for accurate biomimicry of vascular beds, but future efforts will be needed to develop and implement protocols for the creation of organ-specific models of healthy and diseased vasculature.

## Integrating physiological fluid flow into vessel-on-a-chip platforms

Of greatest relevance to this review, vessel-on-a-chip models enable study of bioengineered blood vessels *under controlled flow*, which promises to significantly advance our understanding of how hemodynamic flow contributes to healthy and diseased vascular biology in intact blood vessels. Thus, vessel-on-a-chip design and implementation demands careful attention to the method of introducing circulating flow, which entails a balance between ease of use and the degree to which researchers wish to mimic the *in vivo* milieu for a given application. This is especially important when considered in the context of the aforementioned “fluid shear setpoint” hypothesis advanced by Baeyens et al. (Baeyens et al., 2016). There is also inherent applied hydrostatic pressure that will vary with the total liquid volume, reservoir height, and layout of the fluidic channel circuit in each vessel-on-a-chip design. Within replicates of a single design exposed to the same fluid flow conditions, the applied hydrostatic pressure is relatively uniform, which allows for isolation of applied shear stress as an experimental variable by varying the flow rate (Alonzo et al., 2015; Lam et al., 2018). Altered hydrostatic pressure should be considered when moving between vessel chip platforms as changes to system parameters such as channel geometry, total liquid volume, and method of generating flow will impart varying hydrostatic pressure profiles.

**FIGURE 3:**
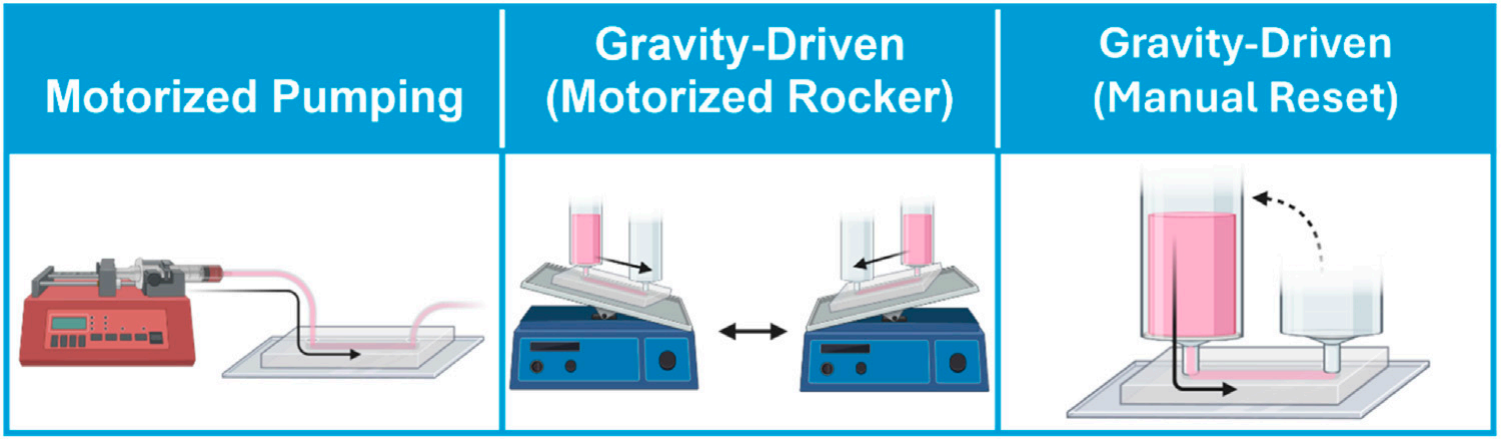
Approaches for circulating flow on vessel-on-a-chip models. Flow is circulated through vessel-on-a-chip platform through motorized pumping, or by gravity-driven flow in which motorized rockers automate hydrostatic pressure reset, or by manual pressure head reset.

Varying WSS experienced by endothelial cells in a vessel-on-a-chip platform is achieved *via* control of applied fluid flow. The primary strategies for controlling fluid flow in vessel-on-a-chip systems include: 1) motorized pumping, 2) motorized rocking of the platform to reset gravity-driven flow, or 3) non-motorized gravity-driven flow with manual reset (Figure 3). Motorized syringe pumps have the advantage of being able to readily achieve high circulating fluid velocity (and thus, correspondingly higher levels of WSS within the ranges seen *in vivo*, Table 1). In addition, motorized pumps (unlike gravity-driven models) allow users to program defined patterns of continuous, oscillatory, and/or pulsatile flow, which may be critical if modeling blood vessel structures in which complex flow profiles are typically observed *in vivo*. However, pump-based systems are mechanically complex, and require numerous accessory components to maintain flow through the pump system. This significantly increases the required volume of circulating media, limits the number of devices that can be run in parallel, and introduces points of potential device failure through fluid leak or microbial contamination. While syringe pumps are often used to provide programmable flow control, peristaltic pumps have become an attractive alternative that offer increased simplicity, lowered cost, and decreased risk of contamination due to the closed nature of recirculating peristaltic systems (Abello et al., 2022; Mohammed et al., 2019; Schneider et al., 2021; Zhang X. et al., 2020; O’Grady et al., 2018). A limitation of such pumps is the inherently pulsatile nature of the produced fluid flow. It is well documented that endothelial cells respond differentially to laminar versus pulsatile flow (Eskin et al., 1984; Helmlinger et al., 1991; Miao et al., 2005; Guo et al., 2007; Mohammed et al., 2019; Liu et al., 2021), thus pulsatile flow patterns will not impart physiological shear forces to small vessels which naturally feature continuous laminar flow in intact microvasculature. Efforts have been made to generate laminar flow *via* peristaltic pumps using pulse dampeners (Voyvodic et al., 2012; Pech et al., 2020; Abello et al., 2022). The transition from pulsatile flow in the macrovasculature to continuous flow in the microvascular beds *in vivo* should be considered when choosing a mechanical pumping approach for specific vessel-on-a-chip applications.

On the other hand, pumpless gravity-driven models are mechanically simpler, and thus potentially easier to scale in studies in which large numbers of devices must be run in parallel (Figure 3). Nonetheless, gravity-driven models also have unique drawbacks. Gravity-driven flow depends upon the hydrostatic pressure gradient generated between two coupled media reservoirs. Thus, gravity-driven flow is initially high when the hydrostatic pressure gradient is largest, but this driving force decreases over time as the volumes of the two reservoirs equilibrate. Not only does this produce a changing flow profile over time, but also necessitates frequent reset of the hydrostatic pressure heads within the two media reservoirs to maintain flow through the system. This process can be performed manually through pipetting, or can be automated using a motorized rocker. Of these two strategies, motorized rockers typically produce bi-directional (i.e., alternating flow direction) gravity-driven fluid circulation, which does not accurately reflect the unidirectionality of blood flow in the intact vasculature. A recent pumpless gravity-driven system corrects this problem using a platform with additional supporting channels and valve features to create continuous unidirectional flow with rocking, although it is not yet in widespread use (Wang and Shuler, 2018). Alternatively, hydrostatic pressure heads can be manually reset through pipetting. While manual pipetting can be tedious, time-consuming, and inconsistent, it is likely that automated robotic pipetting will solve this problem for high-throughput applications, particularly in industrial settings (Langer and Joensson, 2020).

In addition to the above challenges, gravity-driven strategies are also typically limited by media reservoir size, which physically constrains the size of the hydrostatic pressure gradient that can be generated by this approach. As a result, it can be challenging to produce sufficiently high fluid velocity to capture high fluid flow and WSS using gravity-driven approaches. Since WSS depends heavily on vessel diameter, pump-driven models in which high fluid flow rates are introduced in large-diameter vessel compartments, the high fluid velocity may still be insufficient to produce physiological levels of WSS in some models. By contrast, the small vessel sizes (e.g., 25–30 µm in diameter) (Fang et al., 2024) that are often achievable in self-assembled microvasculature-on-a-chip models can bring even gravity-driven models into physiological ranges of WSS (Fang et al., 2024). Precise control of hemodynamic flow, regardless of the mode in which it is applied, in vessel-on-a-chip models is also constrained by the lack of widely adaptable tools for the study of real-time fluid flow. Precise measurement of flow rates and local fluid velocities within a 3D vascular architecture will be necessary for robust quality control and precise determination of WSS effects in functional assays. Fluid velocity can be measured in vessel-on-a-chip models by particle velocimetry, i.e., time-lapse imaging of perfused fluorescent particles from which particle trajectories and velocities can be used to calculate flow patterns and estimate applied WSS based on defined vessel geometry (Pitts and Fenech, 2013). Alternatively, fluid velocity and WSS can be estimated using *in silico* modeling based on the size and shape of bioengineered vessels (Fang et al., 2024; Hatch et al., 2024) using approaches similar to have been previously applied in intact tissue (Bernabeu et al., 2014), but this still presupposes that the pumping or rocking method used generates the desired flow rates within an engineered vessel or vascular bed. Empirical and computational approaches to quantify applied WSS will likely be combined in industrial settings, but these strategies currently present challenges to implementation in most research labs that use vessel-on-a-chip systems. Developing new tools for adaptable and scalable automation of real-time flow monitoring in MPS with complex 3D vasculature will enable modeling of hemodynamics *in vitro* with unprecedented biomimicry.

Another issue for vessel-on-a-chip models is endothelial cell source, particularly with regard to capturing tissue-specific endothelial cell mechanosensitive signaling. Early vessel-on-a-chip models including many described in this review rely on “generic” primary human endothelial cells selected for their ease-of-use and non-specific endothelial cell identity (e.g., human umbilical vein endothelial cells (HUVEC); circulating endothelial progenitor cells (EPC), *etc.*), with the idea that these “generic” endothelial cells would acquire more specialized identities when co-seeded with tissue-specific stromal cells in organ chip platforms. However, with recent studies further underscoring the broad heterogeneity of vascular endothelial cells (Kalucka et al., 2020)—with likely important implications for heterogeneity in mechanosensitive signaling—recent organ chip work has increasingly focused on integrating tissue-specific primary endothelial cells into organ chip settings, with these endothelial cells either isolated directly from tissue-specific vascular beds (e.g. (Park et al., 2019)) or generated from iPSC (e.g. (Vatine et al., 2019)). These latest efforts are more likely to recapitulate tissue-specific flow-sensitive signaling relative to platforms that were developed using “generic” endothelial cells. However, tissue-specific endothelial cells may lose their specialized endothelial cell identities with time in culture, and/or may have compromised vessel self-assembly properties that may limit their integration into platforms that rely upon this property to form vessel-on-a-chip networks. Ongoing work in the field is focused on addressing these various challenges.

A final consideration when incorporating physiological flow into vessel-on-a-chip platforms is the composition of the circulating media. Current vessel-on-a-chip models typically circulate growth factor-rich endothelial growth medium. However, endothelial growth medium often contains higher levels of pro-angiogenic growth factors and other supplements than is typically found in circulating blood. This alone would be hypothesized to drive endothelial cells in vessel-on-a-chip platforms towards a more activated, pro-inflammatory, and pro-angiogenic state more representative of disease, whereas endothelial cells in established and healthy (i.e., uninjured) blood vessels are typically non-proliferative and quiescent (Langille et al., 1986). In addition, the cell culture media typically circulated through vessel-on-a-chip models lacks the higher viscosity of blood and colloidal properties imparted by circulating blood cells—both properties that greatly influence vascular WSS experienced *in vivo*. Additional work remains to be done to improve our current “blood surrogates” to better mimic the biophysical and biochemical properties of *in vivo* blood (Marx et al., 2016). This will in turn further enhance the physiological relevance of circulating fluid flow in vessel-on-a-chip platforms to better recapitulate the effect of hemodynamic forces on intact vasculature.

## Conclusion

Vascularized MPS (i.e., vessel-on-a-chip systems) are powerful *in vitro* models that enable precise control of cell type composition, 3D tissue architecture and layering, extracellular matrix composition, and intravascular fluid flow that regulates biotransport processes and mechanical forces such as applied shear stresses. Current vessel-on-a-chip systems take varied approaches to model the *in vivo* vascular niche, and have successfully recapitulated many of the hallmarks of intact blood vessels that remain elusive in conventional 2D cell culture, while still retaining many of the ease-of-use advantages of *in vitro* experimentation. Balancing complexity and ease-of-use by the widest range of possible end users will be a critical dimension of efforts to maximize translational impact of vessel-on-a-chip technologies. Studies using vessel-on-a-chip models have already produced novel insights into the role of fluid flow and WSS as key signals in vascular health and disease. However, efforts to fully capture the physiological hemodynamics in vascularized MPS remain challenging due to engineering considerations such as controlling and monitoring intravascular fluid flow. Future vessel-on-a-chip platforms must continue to consider how important parameters that determine WSS—including vessel diameter, flow velocity, and fluid viscosity—are represented in their chip designs to ensure that resulting on-chip flow fully captures the biomechanical properties of circulating blood and its corresponding signaling to regulate vascular physiology and function.
